# Cartilage and Bone Serum Biomarkers as Novel Tools for Monitoring Knee Osteochondritis Dissecans Treated with Osteochondral Scaffold

**DOI:** 10.1155/2018/9275102

**Published:** 2018-12-23

**Authors:** Elena Gabusi, Francesca Paolella, Cristina Manferdini, Laura Gambari, Elizaveta Kon, Giuseppe Filardo, Erminia Mariani, Gina Lisignoli

**Affiliations:** ^1^IRCCS Istituto Ortopedico Rizzoli, SC Laboratorio di Immunoreumatologia e Rigenerazione Tissutale, Via di Barbiano 1/10, 40136 Bologna, Italy; ^2^IRCCS Istituto Ortopedico Rizzoli, Laboratorio RAMSES, Via di Barbiano 1/10, 40136 Bologna, Italy; ^3^Humanitas University Department of Biomedical Sciences, Via Manzoni 113, 20089 Rozzano, Milan, Italy; ^4^Humanitas Clinical and Research Center, Via Manzoni 56, 20089 Rozzano, Milan, Italy; ^5^Clinica Ortopedica e Traumatologica I, Laboratorio NABI, IRCCS Istituto Ortopedico Rizzoli, Via di Barbiano 1/10, 40136 Bologna, Italy; ^6^DIMEC, Alma Mater Studiorum Università di Bologna, Via Massarenti 9, 40138 Bologna, Italy

## Abstract

Knee osteochondritis dissecans (OCD) is a focal disease of the joint characterized by modifications of bone and cartilage tissues. Biomimetic osteochondral scaffolds are used to restore these tissues. The aim of this prognostic prospective cohort study was to evaluate serum biomarkers of cartilage (fragments or propeptide of type II collagen: CTXII, C2C, and CPII) and bone (tartrate-resistant acid phosphatase (TRAP) 5b and osteocalcin (OC)) turnover during follow-up of patients treated with an osteochondral scaffold, to identify which were related to healing outcome and clinical score. We found that cartilage (CPII) and bone (OC) synthetic biomarkers were significantly increased during the first-year follow-up, while the respective degradative markers (CTXII, C2C, and TRAP5b) were not modulated. Only CTXII/CPII and C2C/CPII cartilage ratios were significantly modulated, evidencing a higher remodeling of cartilage compared to bone tissue. Cartilage and bone single biomarkers or ratios at one-year follow-up showed values close to or similar to those of healthy subjects. International Knee Documentation Committee (IKDC) score significantly increased from T0 to T2, while the Tegner score did not. Taking into consideration an IKDC score > 70 as clinical success, we found that all OCD cases with both CPII (> 300 pg/ml) and C2C/CPII (<0.35) presented IKDC scores of clinical success. OCD patients treated with an osteochondral scaffold showed an improvement at one-year follow-up, evidenced by both clinical and serum cartilage biomarkers. These data confirmed that cartilage and bone remodeling took place and showed that systemic biomarkers represent a sensitive tool for monitoring OCD patients during the follow-up.

## 1. Introduction

Osteochondritis dissecans (OCD) is a focal idiopathic disease of the joints with unknown etiology, mainly characterized by progressive changes in both cartilage and bone structures. Repetitive microtrauma and ischemia, genetic factors, and growth alteration significantly contribute to OCD development. Patients suffering from OCD show various symptoms such as softening and frequent swelling associated with a partial or complete osteochondral detachment which can cause pain and difficulty in movement and requires clinical treatment [1–3]. Different types of surgical procedures (microfractures, osteochondral autograft, debridement, and scaffolds) are used for knee OCD lesions, not suitable of conventional treatment (use of brace; limitation of activities), which are mainly directed to prevent the development of osteoarthritis (OA)[4, 5].

OCD of the knee is also divided into juvenile and adult forms depending on the maturity of the distal femoral physis (open or close epiphysis) [3, 6]. Magnetic Resonance Imaging (MRI) and radiographic evaluations are routinely used to accurately diagnose OCD lesions. To further assess cartilage and bone modifications, different studies have used histological and immunohistochemical analysis on samples collected from patients during surgical treatment [2, 7–9].

Histological examination of mild OCD showed degeneration accompanied by irregularity and fibrosis in the external layer of cartilage and the presence of necrosis and fibrous tissue in the bone trabeculae [10, 11]. By contrast, severe OCD lesions display increase cell cloning associated with fibrillation/erosion in the articular cartilage and presence of necrotic bone osteoclasts or osteoid areas with disorganized trabeculae [12]. Moreover, immunohistochemical evaluation of cartilage and bone tissue showed the presence of mesenchymal progenitor cells in both cartilage and bone, as well as a high percentage of CD34 and TRAP-positive cells in the bone mainly associated with a high tissue turnover [13]. Nevertheless, these immunohistochemical evaluations do not help to establish the cartilage and bone modifications after patient treatment.

It has been shown that systemic biochemical markers of cartilage and bone turnover can be used to perform early diagnosis or monitor the progression of joint lesions. The main cartilage degradation molecules studied as biomarkers are cleavage of type II collagen (C2C), amino-terminal neoepitopes generated by cleavage of native human type II collagen (Col2-1(4N1)), type II collagen one-quarter fragment secondary collagenase cleavage site amino-terminal neoepitope (Col2-1(4N2)), C1,2C, and C-telopeptide of type II collagen (CTXII), originated from the proteolysis of collagen type II and the principal component of the cartilage [14–19].

Procollagen II C-propeptide (CPII) and serum N-propeptide of collagen IIA (PIIANP) biomarkers of cartilage synthesis are also widely evaluated to define the remodeling of this tissue [15, 20, 21]. Bone biomarkers mainly used to evaluate bone degradation are tartrate-resistant alkaline phosphatase TRAP5b, cross-linked telopeptides of collagen I, and hydroxyproline. TRAP5b is an isoform made up of a group of lysosomal enzymes produced by osteoclasts, which produces reactive oxygen species to digest bone degradation products in the microenvironment of the bone matrix. Bone synthetic biomarkers are osteocalcin (OC), procollagen type I N-terminal (PINP), and alkaline phosphatase (ALP). OC is the most abundant noncollagenous bone protein synthesized by osteoblast, able to bind to hydroxyapatite crystals involved in calcium binding. Nowadays, it is considered as a specific marker of osteoblast functions [20, 22, 23].

To gain new insight in the evaluation of cartilage and bone remodeling during follow-up after surgical treatment, we evaluated OCD patients treated with a cell-free biomimetic scaffold that simulates the osteochondral anatomy [24]. Biomarkers of cartilage and bone turnover were detected in serum samples during follow-up and correlated with clinical scores: the International Knee Documentation Committee (IKDC) and Tegner scores [25, 26]. We demonstrated that at one-year follow-up, mainly cartilage biomarkers increased, suggesting that the remodeling process occurred earlier in the cartilage tissue than in bone.

## 2. Material and Methods

### 2.1. Patient Characteristics

In this study, 14 OCD patients were included, presenting focal lesions (ranging from 1.5 cm^2^ to 4 cm^2^) of the articular surface (no evidence of other chondral-osteochondral, ligament, meniscus, or synovial lesions), with stable and physiologically aligned knees. X-ray and MRI surgical indications were confirmed intra-articularly, and patients were staged as grade 3 OCD lesions, according to the ICRS evaluation package, [https://www.secot.es/uploads/descargas/formacion/escalas_valoracion/ICRS._TRAUMA_CARTaILAGO.pdf]. This evaluation includes the International Knee Documentation Committee (IKDC) Knee Examination-form (established in 2000) administered to assess symptoms and function in daily living activities. All patients were surgically treated with the implantation of a biomimetic osteochondral scaffold (MaioRegen®, Fin-Ceramica, Faenza SpA, Italy). The IKDC scores were collected at three time points: at baseline, before operation (T0), and at 3 months (T1) and at 1 year (T2) after the intervention. The characteristics of each patient included in the study are summarized in Table 1. All subjects prior to injury had a normal level of working (10 patients) or sport activity (4 patients) and showed a Tegner score (from 0 = invalid to 10 = agonistic activity) of 2.8 ± 0.6 (mean ± SD) at the time of clinical intervention. The study was approved by the local ethical committee and all patients signed a written informed consent form prior to inclusion.

### 2.2. Serum Collection

Blood samples from OCD patients were collected at T0, T1, and T2 in tubes containing microsilica particles inside and an integrated separator in the lower part of the tube. The samples were immediately centrifuged at 2500xg for 15 minutes and the serum was collected and stored at -80°C. Serum samples from 9 anonymous healthy subjects (mean age 28.5 ± 4.8, sex: 4 female and 5 male, and BMI 22.02 ± 2.54) with no history of injury or surgical intervention before or during the last 4 years were collected.

### 2.3. Biomarkers Evaluation

For cartilage turnover analysis, collagen type II cleavage (C2C, IBEX Pharmaceuticals Inc., Montreal, Québec, Canada) and cross-linked C-telopeptide of type II collagen (CTXII, Elabscience Biotecnology, Park, WuHan, China) were selected as indicators of cartilage degradation, and procollagen II C-propeptide (CPII, IBEX Pharmaceuticals Inc., Montreal, Québec, Canada) was selected as indicator of cartilage synthesis (Table 2).

For bone turnover analysis, tartrate-resistant acid phosphatase active isoform 5b (TRAP5b, QUIDEL, San Diego, CA, USA) was selected as indicator of bone degradation and osteocalcin (OC, QUIDEL, San Diego, CA, USA) as indicator of bone synthesis (Table 2). The serum concentration of analyzed markers was evaluated using a specific Enzyme-Linked Immunoassay (ELISA) method according to manufacturer's instruction.

### 2.4. Statistical Analysis

The normal distribution of continuous data was analyzed with the Kolmogorov-Smirnov test and homogeneity of variances was analyzed by the Levene's test; since the data were not normal and the variances were not homogeneous, nonparametric tests were subsequently used. Statistical analysis for comparing IKDC score, CTXII, C2C, CPII, TRAP5b, OC, CPII/C2C, CPII/CTXII, and OC/TRAP5b at different time points (T0, T1, T2) was performed using the General Linear Model (GLM) with time as fixed effect and patients as random effects; the Sidak test was used as post hoc pairwise analysis. Data were expressed as mean with 95% confidence interval. Statistical analysis for comparing two groups was performed with the Mann-Whitney U test for unpaired data evaluated by exact methods for small samples.

Spearman Rank correlations were performed between clinical (IKDC subjective score) and biological markers. All statistical analysis was performed using SPSS v.19.0 (IBM Corp., Armonk, NY, USA). All results were considered significant for p<0.05.

## 3. Results

### 3.1. Clinical Improvement of OCD Patients after Osteochondral Scaffold Treatment

**Figure 1:**
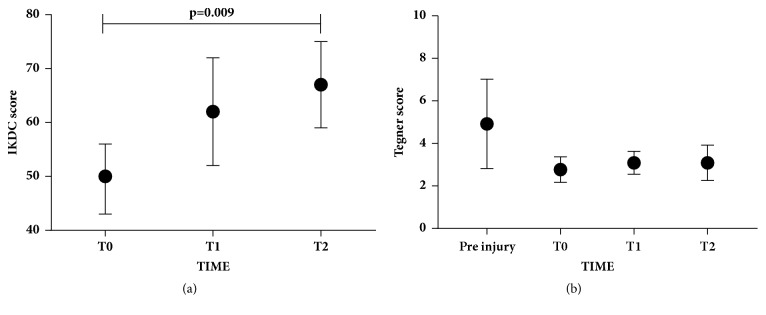
**IKDC subjective score and Tegner score.** (a) IKDC at three different time points (T0= basal, T1= 3 months, and T2= 1 year). Data were expressed as mean ± SD. (b) Tegner score at three different time points (T0= basal, T1= 3 months, and T2= 1 year). Preinjured scores were also included. Data were expressed as mean ± SD. Significant results are indicated.

OCD patients with focal osteochondral knee lesions were treated with an osteochondral scaffold and evaluated with clinical IKDC score at different time points (basal = T0, 3 months = T1, 1 year = T2). As shown in Figure 1(a), IKDC score progressively increased starting from T0 to T2. In particular, a significant improvement was evidenced (p=0.009) from T0 (IKDC score, mean=49.5) to T2 (IKDC score, mean=67.2). As shown in Figure 1(b), after one-year follow-up, the Tegner score did not show a significant increment and was lower than the preinjury score.

### 3.2. Cartilage and Bone Biomarkers of Remodeling are Modulated after Osteochondral Scaffold Treatment

**Figure 2:**
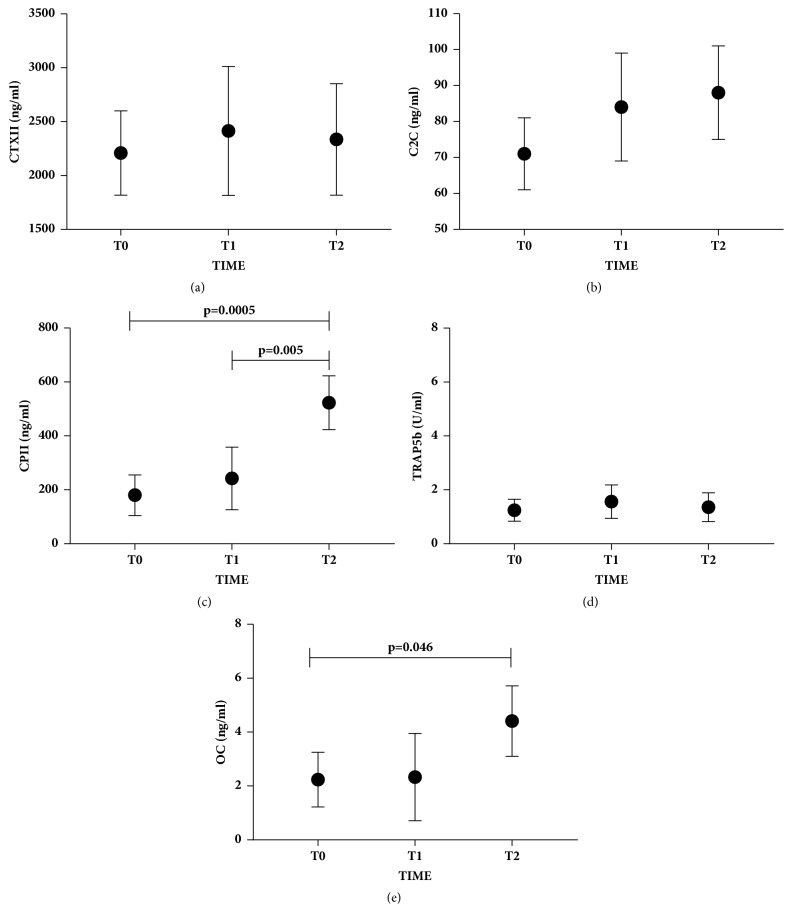
**Serum biomarkers evaluation in OCD during the follow-up. **Biomarkers of cartilage degradation (CTXII; C2C) and synthesis (CPII) and of bone degradation (TRAP5b) and synthesis (OC) at three different time points (T0= basal, T1= 3 months, and T2= 1 year) are represented as mean ± SD. CTXII, C2C, CPII, and OC were expressed as ng/ml while TRAP5b as U/ml. Significant results are indicated.

The serum levels of both cartilage (CTXII, C2C, and CPII) and bone (TRAP5b and OC) biomarkers were measured during the follow-up (T0-T2) of OCD patients treated with the osteochondral scaffold. The two markers of cartilage degradation (CTXII and C2C) were not modulated during the time points evaluated (Figures 2(a) and 2(b)). By contrast, as shown in Figure 2(c), the CPII marker of cartilage synthesis significantly increased starting from T1 until T2 (p=0.005) and from T0 to T2 (p=0.0005). TRAP5b bone biomarker of degradation did not show any modulation at any of the time points considered (Figure 2(d)). By contrast, the OC marker of bone synthesis showed a significant increase from T0 to T2 (p=0.046) (Figure 2(e)).

### 3.3. Cartilage Biomarkers Ratio Modulated during Follow-Up

**Figure 3:**
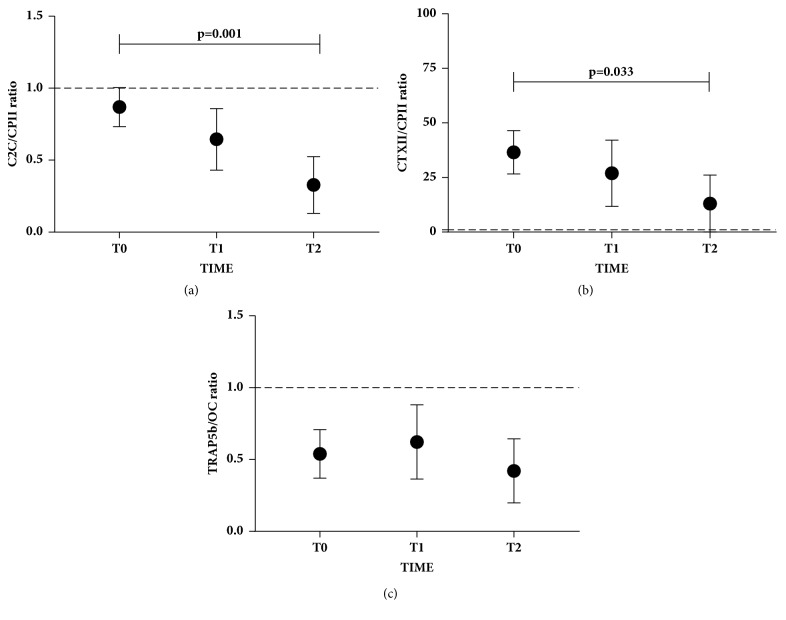
**Cartilage and bone biomarkers ratio during the follow-up. (a)-(b) **CTXII/CPII and C2C/CPII cartilage ratio at three different time points (T0= basal, T1= 3 months, and T2= 1 year) are represented as mean ± SD. Dotted line at 1 represents the point of equilibrium between synthesis and degradation.** (c) **TRAP5b/OC bone ratio at three different time points (T0= basal, T1= 3 months, and T2= 1 year) is represented as mean ± SD. Dotted line at 1 represents the point of equilibrium between synthesis and degradation. Significant results are indicated.

To define the cartilage and bone turnover during follow-up, the following ratios were evaluated: C2C/CPII, CTXII/CPII, and TRAP5b/OC. Considering that value 1 indicates a balance between tissue degradation and synthesis, we found that the C2C/CPII ratio (Figure 3(a)) values were lower than 1 (indicating an active cartilage synthesis) already at T0 and significantly decreased until T2 (p=0.001). On the other hand, at T0, CTXII/CPII ratio (Figure 3(b)) showed values higher than 1 (indicating an active cartilage degradation), which after patient treatment, gradually and significantly (p=0.033) decreased from T0 to T2. By contrast, TRAP5b/OC ratio (Figure 3(c)) showed values lower than 1 (indicating an active bone synthesis), which were not significantly modulated during follow-up.

### 3.4. Biomarkers at End Stage of Follow-Up Showed a Value Close to Those of Healthy Subjects

**Figure 4:**
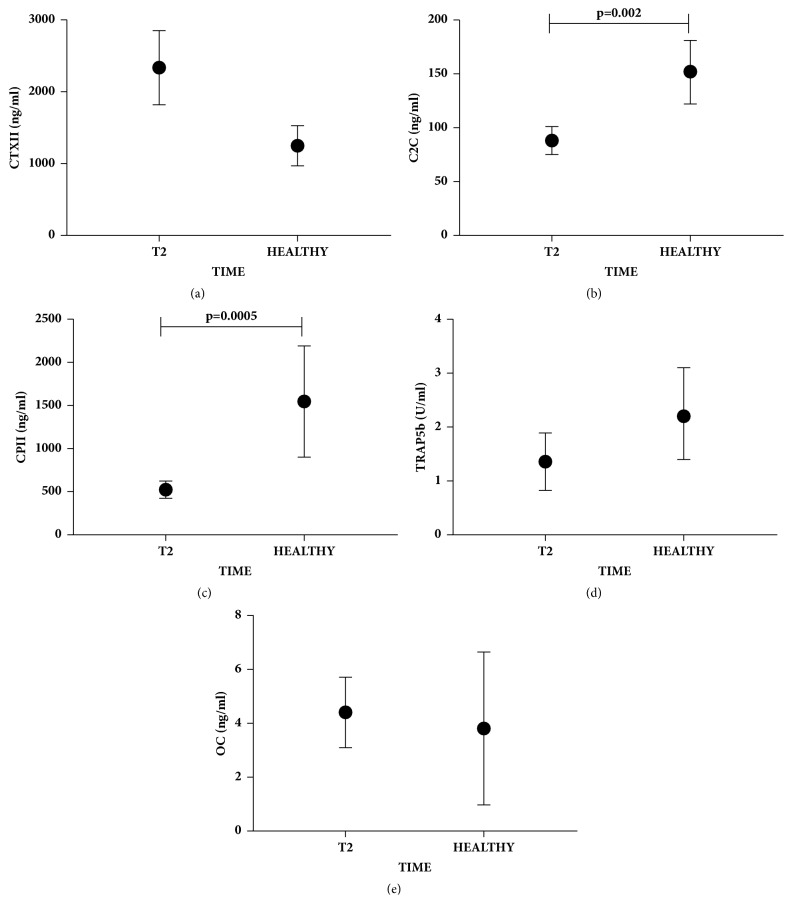
**Serum biomarkers evaluation in OCD patients at one-year follow-up and healthy subjects. **Biomarkers of cartilage degradation (CTXII; C2C), synthesis (CPII), and bone remodeling (TRAP5b) at one-year treated (T2) OCD patients and healthy subjects. Data are expressed as mean ± SD. CTXII, C2C, CPII, and OC were expressed as ng/ml while TRAP5b as U/ml. Significant results are indicated.

**Figure 5:**
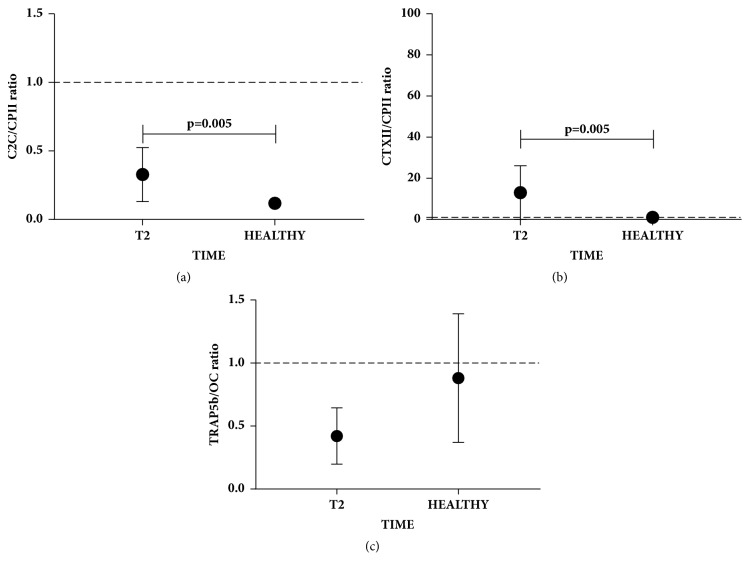
**Cartilage and bone biomarkers ratio in OCD patients at one-year follow-up and healthy subjects. (a)-(b) **CTXII/CPII and C2C/CPII cartilage ratio at 1 year treated (T2) OCD patients and healthy subjects. Data are expressed as mean ± SD. Dotted line at 1 represents the point of equilibrium between synthesis and degradation.** (c) **TRAP5b/OC bone ratio at 1 year treated (T2) OCD patients and healthy subjects. Data are expressed as mean ± SD. Dotted line at 1 represents the point of equilibrium between synthesis and degradation. Significant results are indicated.

Serum levels of cartilage and bone biomarkers of OCD treated patients at T2 were compared with serum level of healthy donors. CTXII, TRAP5b, and OC (Figures 4(a), 4(d), and 4(e), respectively) at T2 reached the same values of healthy donors. By contrast, C2C and CPII biomarkers (Figures 4(b), and 4(c), respectively) were significantly lower in OCD patients (p=0.002 and p=0.0005, respectively). However, as shown in Figures 2(b) and 2(c), these markers increased in the one-year follow-up, reaching values close to those of healthy donors. Moreover, C2C/CPII and CTXII/CPII ratios (Figures 5(a), and 5(b)) were significantly higher in OCD patients (p=0.005 and p=0.005, respectively). However, as shown in Figures 3(a) and 3(b), these ratios decreased in the one-year follow-up, reaching values close to those of healthy donors. TRAP5b/OC ratio was not significantly different from healthy donors.

### 3.5. Combination of CPII and C2C/CPII Ratio Biomarkers Correlate with IKDC Score

**Figure 6:**
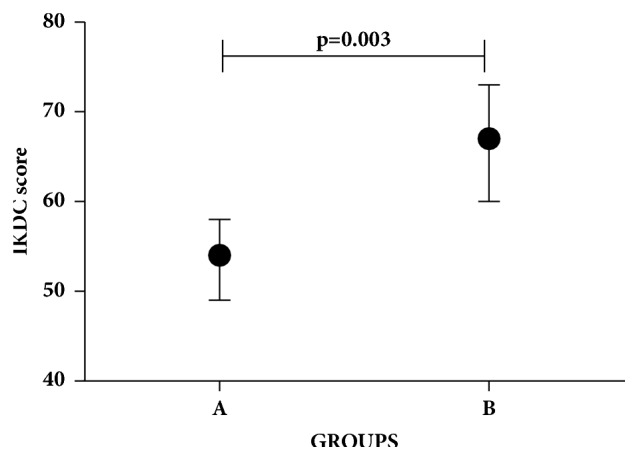
**Combination of CPII and C2C/CPII ratio biomarkers is well indicators of IKDC score. **OCD treated patients that present both CPII higher than 300 ng/ml and C2C/CPII ratio lower than 0.35 (Group B) showed high IKDC score while OCD patient with CPII lower than 300 ng/ml and C2C/CPII ratio higher than 0.35 (Group A) shows a worst IKDC score. Data are expressed as mean ± SD. Significant results are indicated.

Considering an IKDC score <50 as clinical failure and > 70 as clinical success, the OCD cases showed a peculiar distribution for the only two correlated markers (CPII* ŋ*^2^ =0.339, p = 0.072; C2C/CPII ratio* ŋ*^2^ = -0.465, p = 0.013). OCD cases with CPII>300 ng/ml did not show clinical failure at the IKDC score; OCD cases with C2C/CPII ratio <0.35 all showed the clinical success IKDC score. When combining CPII with C2C/CPII ratio, two groups were identified: (1) group A with CPII<300 ng/ml or C2C/CPII ratio >0.35; (2) group B with CPII>300 ng/ml and C2C/CPII ratio <0.35. As shown in Figure 6, group B showed a highly significant clinical success IKDC score (p=0.003). Finally, all healthy subjects had CPII>300 ng/ml and C2C/CPII ratio <0.35 as observed for OCD patients group B.

## 4. Discussion

OCD is a knee osteochondral defect of unknown etiology that involves subchondral bone and articular cartilage that can spontaneously heal or cause instability or detachment of a small and overlying articular cartilage [2]. To date, there is consensus on surgical treatment for OCD lesions, based on the dimension of the lesion and stability. If not appropriately treated, unstable or detached OCD fragments might progress to OA. The management of OCD treatment would benefit from the identification of biomarkers of bone or cartilage remodeling, which can help surgeons in the objective evaluation of the follow-up of these patients [20]. In this series, OCD patients with focal knee lesion (< 4cm^2^) were surgically treated with cell-free biomimetic osteochondral scaffold that restores both bone and cartilage in one surgical step [5]. During one-year follow-up, the systemic serum biomarkers of cartilage and bone remodeling and clinical IKDC and Tegner scores were evaluated. This prognostic prospective cohort study evaluated predictive serum bone/cartilage biomarkers to define which could help to identify the healing outcome of cartilage and bone in OCD patients.

This study confirmed a significant increase of the IKDC score for focal knee lesion of < 4cm^2^ treated with a biomimetic osteochondral scaffold, starting from T0 up to one-year of follow-up. The regeneration of both the cartilaginous and bone tissues could be mainly due to the presence of progenitor cells in the two compartments, as shown in a previous study that demonstrated the presence of mesenchymal progenitor cells in OCD osteochondral fragments that were positive for CD146, both in cartilage and in bone tissues [13]. Interestingly, even after one year, these patients show a significant increase of IKDC score and have started to resume their normal physical activities (Tegner score), confirming that the remodeling process is still in progress [5, 24].

The evaluation of cartilage (CTXII, C2C, and CPII) and bone (TRAP5b and OC) serum biomarkers at the 3 different time points (basal, after 3 months, and 1 year) evidenced that only the synthetic biomarkers of both cartilage (CPII) and bone (OC) significantly increased, while the degradative biomarkers of cartilage (CTXII; C2C) and bone (TRAP5b) were not modulated during the one-year follow-up, suggesting the potentiality of both tissues in the remodeling processes. It has been shown that, during the progression of OA the degradative cartilage, biomarkers increased with the severity of the knee OA. It is known that these systemic biomarkers well reflect the synovial fluid concentration in the knee, indicating that they are good predictors of the healing evolution [27]. The analysis of degradative/synthetic biomarkers ratio of cartilage and bone, respectively, during the follow-up suggests that cartilage remodeling is more efficient and probably occurs earlier than in bone tissue, showing that in OCD healing bone tissue (even if in progress) may be at least, or even more challenging as cartilage. In particular, CTXII/CP2 and C2C/CPII cartilage ratios gradually decreased until the one-year follow-up, but CTXII/CP2 reached value 1, indicating equilibrium between cartilage degradation and synthesis, while C2C/CPII is under value 1, confirming an active synthesis. By contrast, the TRAP5b/OC bone ratio, even if lower than 1, did not show a significant modulation at the one-year follow-up, confirming that cartilage modification occurred earlier. In line with this data, a recent work by Perdisa et al., in a 5-year follow-up study in OCD patients treated with the same osteochondral scaffold, confirmed with MRI observation of cartilage repair tissue (MOCART) that cartilage parameters reached a stable status at approximately 2-year follow-up, while MRI evidenced that subchondral bone at 5-year follow-up was still under remodeling [5, 28].

These results on biomarkers of bone and cartilage remodeling are of interest since OCD, which is considered an osteochondral disease and seems to have significant tissues remodeling at all-time points considered, but are mainly cartilage tissue, as previously reported [5]. Different studies have shown that modulation of type II collagen degradative biomarkers as both C2C and CTXII is directly associated with major cartilage degradation turnover [15, 21]. By contrast, CPII marker is directly correlated with collagen synthesis, since after damage to cartilage, it is produced by chondrocyte and its serum levels are associated with early cartilage lesions [15].

By comparing serum level biomarkers of OCD patients with healthy subjects, we observed that only C2C and CPII markers at one-year follow-up were lower in comparison to healthy subjects. These markers, even if lower than those of healthy subjects, showed a positive trend versus the values of healthy subjects during the time points analyzed. When comparing the ratios of cartilage biomarkers C2C/CPII and CTXII/CPII between OCD patients (at one-year follow-up) and healthy subjects, a positive trend versus the ratio values of healthy subjects was observed, indicating that the scaffold treatment contributed to a significant remodeling process. The single markers of bone tissue synthesis and degradation and respective ratio did not show significant differences with the healthy subjects, thus confirming that cartilage tissue had an earlier remodeling process than bone tissue in OCD patients at one-year follow-up, in line with MRI evaluation of cartilage and bone tissue [5]. Finally, CPII biomarker and C2C/CPII ratio were good indicators of the IKDC score trend. In fact, OCD treated patients with both CPII higher than 300 ng/ml and C2C/CPII ratio lower than 0.35 showed high IKDC score, while OCD patients with CPII lower than 300 ng/ml and C2C/CPII ratio higher than 0.35 showed a worse IKDC score.

In conclusion, these data confirmed, at one-year follow-up, that cartilage biomarkers represent an efficient quantitative and objective method to evaluate the positive or negative evolution of OCD patients after treatment with osteochondral scaffolds. Extended follow-up of OCD patients treated with osteochondral scaffold can be useful to confirm this trend, the potentiality of bone biomarkers, and to compare this data with imaging evaluations. Indeed, serum biomarkers of cartilage have potential utility in monitoring and assessing tissue regeneration in OCD patients, since they are fairly easy to collect in serum and are less expensive and sensitive to evaluate the outcome of OCD patients.

## Figures and Tables

**Table 1:** Patients' characteristics.

**Number of Patients (n.)**	14 (4 athletes)
**Lesion location**	1 trochlea
	13 MFC

**Sex**	4 Female
	10 Male

**Age (mean ± SD)**	23.6 ± 8.6

**BMI (mean ± SD)**	23.99 ± 4.24

**Physes**	14 closed

MFC= medial femoral condyle; BMI = Body Mass Index.

**Table 2:** Serum biomarker characteristics.

**SERUM BIOMARKERS**	**NAME**	**INVOLVEMENT**
**C2C**	Human Collagen type II cleavage	Cartilage degradation

**CTXII**	Human Cross-Linked C-Telopeptides of Type II Collagen	Cartilage degradation

**CPII**	Human Procollagen II C-propeptide	Cartilage synthesis

**TRAP5b**	Human Tartrate Resistant Alkaline Phosphatase	Bone degradation

**OC**	Human Osteocalcin	Bone synthesis

## Data Availability

The data used to support the findings of this study have not been made available because no specific permission was requested to the ethical committee for data availability.

## Abbreviations

ALP: Alkaline phosphatase
Col2-1(4N1): Neoepitopes generated by cleavage of native human type II collagen
COL2-1/4N2: Type II collagen one-quarter fragment secondary collagenase cleavage site amino-terminal neoepitope
CPII: Procollagen II C-propeptide
CTXII: C-telopeptide of type II collagen
C2C: Cleavage of type II collagen
GLM: General Linear Model
IKDC: International Knee Documentation Committee
MRI: Magnetic Resonance Imaging
OC: Osteocalcin
OCD: Osteochondritis dissecans
PIIANP: Serum N-propeptide of collagen IIA
PINP: Procollagen type I N-terminal propeptide
TRAP5b: Tartrate-resistant acid phosphatase 5b.
